# High serum uric acid level is a mortality risk factor in peritoneal dialysis patients: a retrospective cohort study

**DOI:** 10.1186/s12986-019-0379-y

**Published:** 2019-08-01

**Authors:** Shilong Xiang, Xiaohui Zhang, Xishao Xie, Junni Wang, Qin Zhou, Zhimin Chen, Yaomin Wang, Guangjun Liu, Fei Han, Jianghua Chen

**Affiliations:** Kidney Disease Center, The First Affiliated Hospital, School of Medicine, Zhejiang University, Hangzhou, 310003 China

**Keywords:** End stage renal disease, Peritoneal dialysis, Mortality, Uric acid

## Abstract

**Background:**

The results remain controversial with regards to the impact of serum uric acid on clinical outcomes from peritoneal dialysis population. The aim of our study was to investigate the influence of serum uric acid levels on mortality in peritoneal dialysis patients.

**Methods:**

Data on 9405 peritoneal dialysis patients from the Zhejiang Renal Data system were retrospectively analyzed. All demographic and laboratory data were recorded at baseline. The study cohort was divided into quintiles according to baseline uric acid level (mg/dL): Q1 (< 6.06), Q2 (6.06–6.67), Q3 (6.68–7.27) (reference), Q4 (7.28–8.03), and Q5 (≥8.04). Hazards ratio (HR) of all-cause and cardiovascular mortality was calculated.

**Results:**

Mean serum uric acid was 7.07 ± 1.25 mg/dL. During a median follow-up of 29.4 (range, 3.0 to 115.4) months, 1226 (13.0%) patients died, of which 515 (5.5%) died of cardiovascular events. The Kaplan-Meier survival curves showed that patients in the middle uric acid quintile (Q3: 6.68–7.27) exhibited the highest patient and cardiovascular survival rates (log-rank test *P* < 0.05). Multivariate Cox regression analysis showed that, using Q3 as the reference, in the fully adjusted model, a higher uric acid level (Q4: 7.28–8.03, and Q5: ≥8.04) was significantly associated with higher all-cause mortality (Model 3; Q4: HR, 1.335, 95% CI, 1.073 to 1.662, *P* = 0.009; Q5: HR, 1.482, 95% CI, 1.187 to 1.849, *P* = 0.001), but not with cardiovascular mortality. The adverse effect of higher uric acid level (≥7.28 mg/dL) on all-cause mortality was more prominent in groups such as male, hypoalbuminemia, normal weight, non-diabetes mellitus at baseline rather than in their counterparts respectively.

**Conclusions:**

A higher uric acid level was an independent risk factor for all-cause mortality in peritoneal dialysis patients.

**Electronic supplementary material:**

The online version of this article (10.1186/s12986-019-0379-y) contains supplementary material, which is available to authorized users.

## Background

Uric acid (UA) is the final product of purine or nucleotide metabolism, about two-thirds of which is excreted by glomerular filtration [1, 2]. An elevated UA level is often encountered in patients with chronic kidney disease (CKD) and end-stage renal disease (ESRD) due to decreased renal function. Apart from the CKD-induced metabolism alteration, the serum UA concentration of ESRD population is also influenced by nutritional state, pharmacological interventions and dialysis treatment [3].

In general population, hyperuricemia has been shown to be associated with high-risk of hypertension [4], peripheral and coronary arterial disease [5, 6], cardiovascular disease [7], diabetes mellitus [8], and CKD [9]. Epidemiological studies have also identified the cardiovascular events and mortality predictive role of higher serum UA levels in CKD patients [10–12]. For dialysis population, it has been equivocal regarding the impact of UA on the outcomes of dialysis patients from current evidence. Studies have revealed a positive, negative, or “J-shaped” relationship between UA and all-cause or cardiovascular mortality in hemodialysis (HD) population [3, 13–16]. With regard to peritoneal dialysis (PD) population, only a handful of studies have indicated inconsistent association between serum UA and outcomes. To date, 2 previous studies including 156 and 985 PD patients respectively demonstrated that elevated UA level was an independent risk factor for cardiovascular and all-cause mortality in PD patients [17, 18], whereas another study of 492 patients demonstrated an inverse association between hyperuricemia and mortality risk [19]. An U-shaped UA level-mortality relationship was found in a longitudinal study of 300 patients [20]. In addition, a large-scale multicenter PD cohort study found a weak prognostic value of UA in cardiovascular and all-cause mortality [21]. Besides, sex-related differences in UA-mortality relationship in PD patients have been reported, and it is said that UA level to be a predictor of mortality in men only [18, 22].

In short, the results on UA and mortality from PD population remain controversial. And almost of these studies above were single-center and small or medium-scale ones. What’s more, there is no specific recommendation given to use agents to lower serum UA levels of CKD patients in the KDIGO guideline, on count of insufficient evidence [23]. And it is not mentioned whether or not to apply drug interventions on hyperuricemia of PD patients in the ISPD guideline [24]. Therefore, the aim of our present study was to explore the association between serum uric acid level and all-cause or cardiovascular mortality among PD patients, as well as to inform treatment decisions in routine clinical practice, through a retrospective analysis of a large-scale multicenter cohort of incident PD patients in China.

## Methods

### Study population

We conducted a retrospective analysis of patient data from the Zhejiang Renal Data system (ZJRDS). ZJRDS was established in 2007 that collects and reports the incidence, prevalence and outcome of dialysis treatment for patients with ESRD in Zhejiang province, China. By the end of 2017, there are 235 HD and 98 PD centers have been registered in this system and updating the data regularly. Cases for our analysis were the incident cohort of ESRD patients solely received PD therapy from 1 January 2008 to 31 December 2016. The algorithm for the cohort definition is shown in Additional file 1: Figure S1. We included patients with the following criteria: (1) who had an age above 18 years at the initiation of PD therapy; (2) who had a follow-up of more than 3 months; and (3) who had at least two or more serum UA measurements within 3 months after the initiation of PD therapy. The exclusion criteria included the following: (1) who had no available serum UA level; and (2) who had a history of HD or renal transplantation. This study was performed in accordance with the Declaration of Helsinki. The study was approved by the Clinical Research Ethics Committee of the First Affiliated Hospital of Zhejiang University.

### Exposure assessment

All patients regularly had outpatient visits at 0.5, 1, 2, and 3 months after PD catheter insertion in Zhejiang province, and then every 1–2 months. The primary exposure of interest was serum UA level averaged over the first 3 months after the initiation of PD therapy. The median number of UA measurements contributed by each patient was 4 (Interquartile range, 3–4). Based on quintile of serum UA level, UA (in mg/dL) was grouped into the five following categories: < 6.06, 6.06–6.67, 6.68–7.27 (reference), 7.28–8.03, and ≥ 8.04. We also examined UA as continuous predictor using fractional polynomial regression models.

### Covariates assessment

Demographic and biochemical data were extracted from the ZJRDS. Demographic characteristics, including sex, age, primary kidney disease, comorbid conditions, such as diabetes and cardiovascular disease (CVD) were collected at the time of PD initiation. CVD was defined as the presence of diagnostic codes for angina, coronary artery disease, myocardial infarction, congestive heart failure, or cerebrovascular disease. Body mass index (BMI) was calculated as bodyweight (kg) divided by squared body height (m^2^). Residual renal function (RRF) was calculated as the mean of the urea and creatinine clearance and adjusted for 1.73 m^2^ of body surface area from a 24-h urine collection. Laboratory measurements encompassed hemoglobin, serum albumin, serum potassium, serum natrium, serum phosphorus, serum calcium, serum parathyroid hormone (PTH), serum creatinine, and fasting plasma glucose level, all of which were examined at monthly or 3-monthly intervals. The measurements of BMI, RRF and laboratory data in the first 3 months were averaged as baseline values.

### Outcome assessment

The primary outcomes of interest were all-cause mortality and cardiovascular mortality. Cardiovascular death was defined as death caused by acute myocardial infarction, atherosclerotic heart disease, arrhythmia, cardiomyopathy, congestive heart failure, or cerebrovascular events [25]. Follow-up time started at the day of PD therapy initiation. Patients were censored at the time of kidney transplantation, transfer to HD, loss to follow-up, and the end of the study (June 30, 2017).

### Statistical analysis

The baseline characteristics are presented according to UA categories. Continuous variables are presented as the means and the standard deviations or the medians with the interquartile ranges, and categorical variables are presented as frequencies with percentages. Differences among the UA groups were examined using analysis of variance and chi-square tests for continuous and categories variables, respectively. To assess the relationship between the serum UA level and demographic and clinical data, univariate and multivariate linear regression analyses were performed.

To explore the association of UA categories with all-cause and cardiovascular mortality, Cox proportional hazard regression models were performed according to the 5 UA groups. Factors that showed a significant association (*P* < 0.10) after univariate analysis (age, BMI, RRF, hemoglobin, albumin, potassium, natrium, phosphorus, calcium, PTH, creatinine, fasting plasma glucose, diabetes, CVD and follow-up duration) or were of considerable theoretical relevance (sex) were entered into the multivariate Cox regression analysis (Additional file 5: Table S2 and Additional file 6:Table S3). We adjusted the models for following potential confounders:(1) model 1: unadjusted; (2) model 2: adjusted for demographic clinical characteristics that included age, BMI, sex; (3) model 3: adjusted for the model 2 covariates and diabetes mellitus, CVD, RRF, hemoglobin, serum albumin, potassium, natrium, phosphorus, calcium, PTH, creatinine, and fasting plasma glucose. A Kaplan-Meier analysis was also used to evaluate the change in survival between the quintiles of UA, and curves were compared using the log-rank test. In addition, we examined the nonlinear associations of continuous UA with all-cause and cardiovascular mortality using fractional polynomial regression models. Furthermore, we conducted the examination of *P* value of interaction between UA and several traditional risk factors. For interactions, *P* < 0.1 was considered significant [26]. Based on the results (Fig. 3), we further divided patients into subgroups age, sex, BMI, and albumin. We also performed subgroup analyses according to DM because it was of important clinical concern and was proven to have a different effect on mortality [21]. A two-tailed *P* < 0.05 was considered statistically significant. All statistical analyses were performed using SPSS, version 20.0 for Mac (SPSS Inc., Chicago, IL) and Stata/MP, version 14.1 (StataCorp LP, College Station, TX).

## Results

### Demographic and baseline characteristics

Among a total of 11335 patients in the ZJRDS cohort, 9405 patients who met the inclusion criteria were included in the final analysis (Additional file 1: Figure S1). Additional file 2: Figure S2 shows the distribution of serum uric acid, whose level being approximately normally distributed, with a mean of 7.07 ± 1.25 mg/dL and a median (interquartile range) of 6.97 (6.23–7.81) mg/dL. The baseline characteristics of the overall cohort and stratified by UA categories are presented in Table 1, with relevant data summarized as follows: mean age, 52.5 ± 14.6 years; 54.9% men; 12.3% with diabetes; mean RRF was 6.76 ± 3.13 ml/min/1.73m^2^; mean baseline creatinine level of 9.6 ± 3.2 mg/dL; and mean follow-up time, 34.5 ± 23.2 months.

**Table 1:** Baseline characteristics according to quintile of serum uric acid level (*n* = 9405)

Variants		Serum uric acid level (mg/dl)						
	Total (n = 9405)	< 6.06 (*n* = 1,865)	6.06–6.67 (n = 1,877)	6.68–7.27 (n = 1,892)	7.28–8.03 (n = 1,878)	≥8.04 (n = 1,893)	*P* Value	N(%)
Age (yr)	52.5 ± 14.6	55.5 ± 15.1	52.7 ± 14.5	51.6 ± 14.5	51.7 ± 14.0	51.0 ± 14.3	< 0.001	9405 (100)
Men (N, %)	5166 (54.9)	822 (44.1)	933 (49.7)	1012 (53.5)	1159 (61.7)	1240 (65.5)	< 0.001	9405 (100)
BMI (kg/m^2^)	21.7 ± 3.2	21.0 ± 3.2	21.4 ± 2.9	21.9 ± 3.1	22.1 ± 3.3	22.4 ± 3.3	< 0.001	8456 (89.9)
Hemoglobin (g/L)	97.6 ± 12.2	97.8 ± 12.2	98.1 ± 11.5	97.6 ± 11.7	97.7 ± 11.5	96.8 ± 13.4	0.013	9393 (99.9)
Albumin (g/L)	35.5 ± 5.3	34.2 ± 5.7	35.4 ± 5.1	35.7 ± 5.0	36.1 ± 5.1	36.2 ± 5.3	< 0.001	9260 (98.5)
Creatinine (mg/dL)	9.6 ± 3.2	8.6 ± 3.0	9.6 ± 3.1	9.8 ± 3.1	9.9 ± 3.3	9.9 ± 3.5	< 0.001	9405 (100)
Calcium (mmol/L)	2.21 ± 0.19	2.21 ± 0.19	2.24 ± 0.18	2.22 ± 0.19	2.22 ± 0.19	2.19 ± 0.20	< 0.001	9334 (99.2)
Phosphorus (mmol/L)	1.59 ± 0.36	1.44. ± 0.36	1.55. ± 0.32	1.61 ± 0.33	1.64 ± 0.34	1.70 ± 0.37	< 0.001	9322 (99.1)
Potassium (mmol/L)	4.21 ± 0.47	4.13 ± 0.49	4.17 ± 0.46	4.24 ± 0.45	4.25 ± 0.46	4.28 ± 0.47	< 0.001	9334 (99.2)
Natrium (mmol/L)	140.37 ± 2.26	140.00 ± 2.48	140.30 ± 2.19	140.49 ± 2.01	140.51 ± 2.18	140.56 ± 2.33	< 0.001	9324 (99.1)
PTH (pg/mL)	304.1 ± 221.3	269.19 ± 207.9	299.6 ± 223.4	325.4 ± 231.9	311.4 ± 221.7	314.0 ± 216.5	< 0.001	9253 (98.4)
RRF (mL/min/1.73m^2^)	6.76 ± 3.13	7.19 ± 3.46	6.60 ± 3.04	6.57 ± 3.01	6.82 ± 3.09	6.67 ± 2.99	< 0.001	7766 (82.6)
AKP (U/L)	80.1 ± 43.9	83.0 ± 42.4	79.3 ± 45.3	78.5 ± 41.0	78.9.1 ± 34.6	81.3 ± 53.4	0.676	1762 (18.7)
Uric acid (mg/dL)	7.1 ± 1.2	5.5 ± 0.5	6.4 ± 0.2	7.0 ± 0.2	7.6 ± 0.2	8.9 ± 0.8	< 0.001	9405 (100)
FPG (mmol/L)	5.8 ± 1.6	6.1 ± 1.9	5.8 ± 1.6	5.7 ± 1.5	5.8 ± 1.5	5.6 ± 1.6	< 0.001	9047 (96.2)
HbA1c(%)	6.5 ± 3.9	6.3 ± 2.5	6.2 ± 2.4	6.6 ± 3.7	6.8 ± 6.0	6.5 ± 3.4	0.029	3250 (34.6)
DM(N,%)	1147 (12.2)	274 (14.7)	242 (12.9)	207 (10.9)	225 (12.0)	199 (10.5)	0.001	9405 (100)
CVD(N,%)	474 (5.0)	114 (24.1)	94 (19.8)	81 (17.1)	97 (20.5)	88 (18.6)	0.115	9405 (100)
Primary kidney disease							< 0.001	
Glomerulonephritis(N,%)	5073 (53.9)	889 (17.5)	995 (19.6)	1045 (20.6)	1038 (20.5)	1106 (21.8)		
Diabetic nephropathy(N,%)	826 (8.8)	196 (23.7)	187 (22.6)	160 (19.4)	161 (19.5)	122 (14.8)		
Renal vascular(N,%)	335 (3.6)	72 (21.5)	59 (17.6)	75 (22.4)	72 (21.5)	57 (17.0)		
Obstructive nephropathy(N,%)	77 (0.8)	14 (18.2)	9 (11.7)	14 (18.2)	21 (27.3)	19 (24.6)		
Polycystic kidney disease (N,%)	133 (1.4)	31 (23.3)	40 (30.1)	19 (14.3)	22 (16.5)	21 (15.8)		
Other(N,%)	2961 (31.5)	663 (22.4)	587 (19.8)	579 (19.6)	564 (19.0)	568 (19.2)		
Follow-up duration (mo)	34.5 ± 23.2	35.2 ± 24.1	37.5 ± 24.2	36.3 ± 23.4	33.7 ± 22.0	29.9. ± 21.3	< 0.001	9405 (100)
All-cause death (N,%)	1226 (13.0)	334 (17.9)	245 (13.1)	185 (9.8)	226 (12.0)	236 (12.5)	< 0.001	9405 (100)
Cardiovascular death(N,%)	515 (5.5)	131 (7.0)	114 (6.1)	82 (4.3)	86 (4.6)	102 (5.4)	0.001	9405 (100)

Note: Values are presented as mean ± standard deviation or number (%)

Abbreviations: *BMI* body mass index, *PTH* Parathyroid hormone, *RRF* Residual renal function, *AKP* alkaline phosphatase, *FPG* fasting plasma glucose, *DM* diabetes mellitus, *CVD* cardiovascular disease

Compared to patients with serum UA level in the lowest quintile, patients with higher serum UA level were more likely to be younger, male and obese, as well as had a lower prevalence of diabetes. Serum albumin, baseline creatinine, phosphorus, potassium, natrium, and PTH levels increased, while RRF and fasting plasma glucose decreased in the higher quintiles. Serum hemoglobin, HbA1c, calcium and alkaline phosphatase (AKP) were not significantly different between the lowest quintile and higher ones.

### Clinical parameters affecting the serum UA level

Next, we explored the baseline clinical parameters affecting the serum UA level in our study population. The results are listed in Additional file 4: Table S1. The serum UA level was positively correlated with BMI, serum albumin, baseline creatinine, phosphorus, potassium, and natrium level. After making an adjustment, we found that men, a higher BMI, higher albumin levels, higher serum phosphorus and potassium levels, lower calcium and fasting plasma glucose levels were significantly associated with higher serum UA levels.

### Survival analyses

**Fig. 1:**
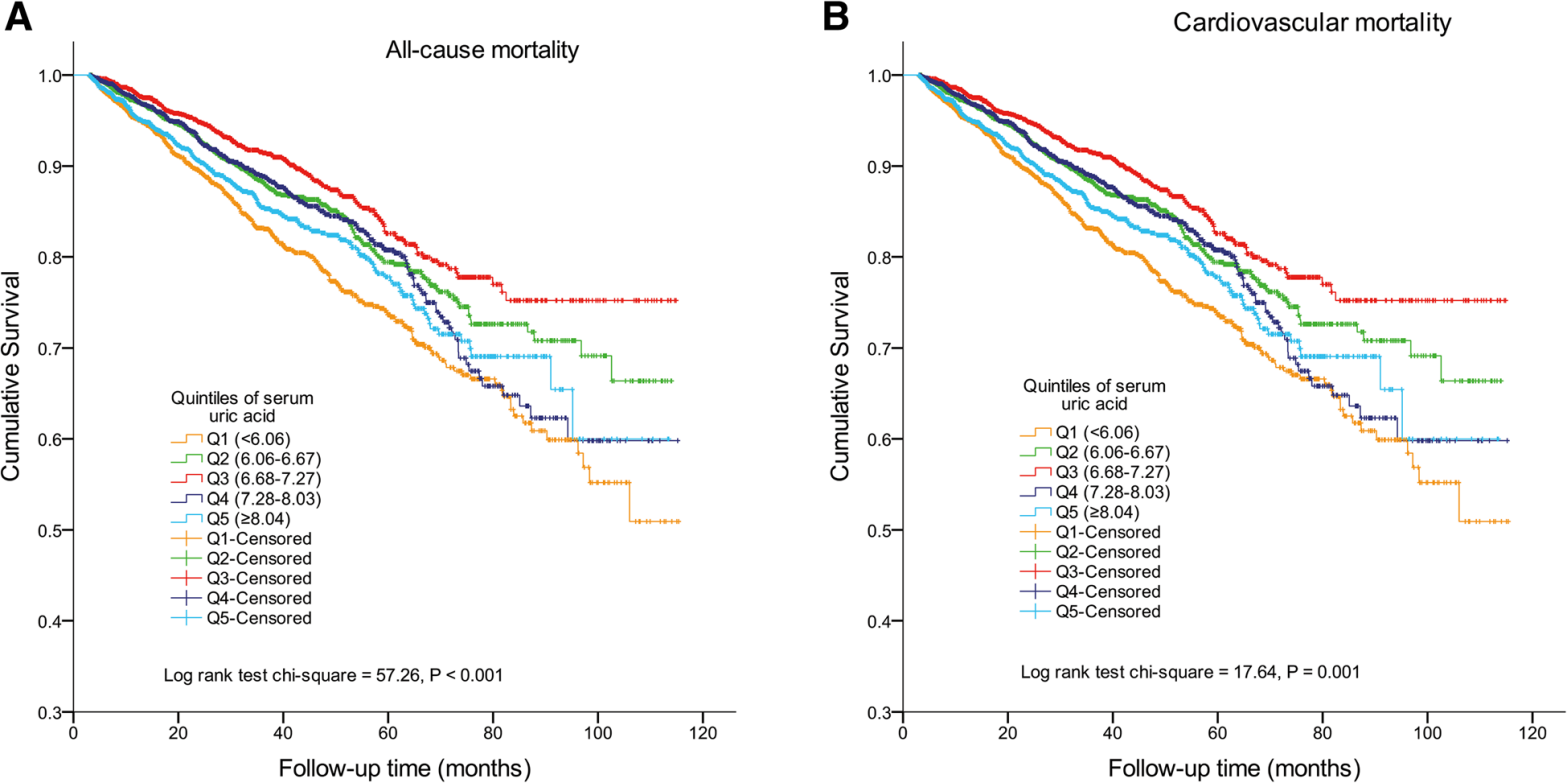
Kaplan-Meier survival curves for quintiles of serum uric acid level in patients undergoing peritoneal dialysis. **a** All-cause mortality and (**b**) cardiovascular mortality. Q1 (< 6.06 mg/dL), Q2 (6.06–6.67 mg/dL), Q3 (6.68–7.27 mg/dL), Q4 (7.28–8.03 mg/dL), and Q5 (≥8.04 mg/dL)

The median follow-up period was 29.4 months (range from 3.0 to 115.4 months). During the study period, 1226 (13.0%) patients died, 1224 (13.0%) patients were transferred to HD, and 708 (7.5%) patients underwent kidney transplantation. Of those, 515 (5.5%) died of cardiovascular events (Table 1). The Kaplan-Meier survival curves for all-cause and cardiovascular mortalities for the patients with the different UA levels are illustrated in Fig. 1. The lower and higher UA level groups significantly increased all-cause and cardiovascular mortality; the patients in the middle UA quintile (Q3: 6.68–7.27) exhibited the highest patient and cardiovascular survival rates, compared with the other 4 UA groups (log-rank test *P* < 0.05).

Tables 2, 3 display the hazard ratios (HRs) of all-cause and cardiovascular mortality that were associated with the different UA levels while considering Q3 group as the reference. According to the multivariable Cox proportional hazards model, after adjusting for various covariates, a higher UA level (Q4: 7.28–8.03, and Q5: ≥8.04) was significantly associated with higher all-cause mortality (Model 3; Q4: HR, 1.335, 95% CI, 1.073 to 1.662, *P* = 0.009; Q5: HR, 1.482, 95% CI, 1.187 to 1.849, *P* = 0.001), but not the lower UA level groups (Model 3; Q1: HR, 1.162, 95% CI, 0.945 to 1.427, *P* = 0.154; Q2: HR, 1.160, 95% CI, 0.938 to 1.434, *P* = 0.172). Whereas cardiovascular mortality was not significantly associated with serum UA level (Model 3; Q1: *P* = 0.292; Q2: *P* = 0.120; Q4: *P* = 0.463; Q5: *P* = 0.482).

**Table 2:** All-cause mortality associated with quintiles of serum uric acid in unadjusted and multivariable-adjusted Cox models

Variants	Event	Unadjusted Model 1	*P* value	Multivariable adjusted Model 2	*P* value	Multivariable adjusted Model 3	*P* value
Quintile of uric acid level (mg/dL)	1226/9405 (13.0)						
Q1(< 6.06)	334/1865 (17.9)	1.888 (1.577–2.259)	< 0.001	1.434 (1.181–1.742)	< 0.001	1.162 (0.945–1.427)	0.154
Q2(6.06–6.67)	245/1877 (13.1)	1.278 (1.056–1.547)	0.012	1.180 (0.962–1.447)	0.112	1.160 (0.938–1.434)	0.172
Q3 (6.68–7.27)	185/1892 (9.8)	Reference		Reference		Reference	
Q4 (7.28–8.03)	226/1878 (12.0)	1.341 (1.104–1.628)	< 0.001	1.253 (1.015–1.547)	0.036	1.335 (1.073–1.662)	0.009
Q5 (≥8.04)	236/1893 (12.5)	1.595 (1.316–1.934)	0.048	1.481 (1.200–1.828)	< 0.001	1.482 (1.187–1.849)	0.001

Note: Reference group is Q3 group (6.68–7.27 mg/dL). Values are presented as number (%) of events or hazard ratio (95% confidence interval). Q, quintile

Model 1 are unadjusted. Model 2 are adjusted for the factors of age, sex, body mass index. Model 3 are adjusted for the factors of model 1 covariates and diabetes mellitus, cardiovascular disease, residual renal function, hemoglobin, serum albumin, serum potassium, serum natrium, serum phosphorus, serum calcium, serum parathyroid hormone, serum creatinine, and fasting plasma glucose

**Table 3:** Cardiovascular mortality associated with quintiles of serum uric acid in unadjusted and multivariable-adjusted Cox models

Variants	Event	Unadjusted Model 1	*P* value	Multivariable adjusted Model 2	*P* value	Multivariable adjusted Model 3	*P* value
Quintile of uric acid level (mg/dL)	408/9405 (4.0)						
Q1(< 6.06)	131/1865 (7.0)	1.667 (1.265–2.197)	<0.001	1.327 (0.986–1.786)	0.062	1.166 (0.820–1.657)	0.392
Q2(6.06–6.67)	114/1877 (6.0)	1.341 (1.010–1.782)	0.043	1.268 (0.942–1.706)	0.118	1.311 (0.932–1.843)	0.120
Q3 (6.68–7.27)	82/1892 (4.3)	Reference		Reference		Reference	
Q4 (7.28–8.03)	86/1878 (4.6)	1.152 (0.851–1.559)	0.359	1.088 (0.791–1.497)	0.602	1.146 (0.796–1.648)	0.463
Q5 (≥8.04)	102/1893 (5.4)	1.555 (1.162–2.080)	0.003	1.259 (0.916–1.731)	0.156	1.144 (0.786–1.665)	0.482

Note: Reference group is Q3 group (6.68–7.27 mg/dL). Values are presented as number (%) of events or hazard ratio (95% confidence interval). Q, quintile

Model 1 are unadjusted. Model 2 are adjusted for the factors of age, sex, body mass index. Model 3 are adjusted for the factors of model 1 covariates and diabetes mellitus, cardiovascular disease, residual renal function, hemoglobin, serum albumin, serum potassium, serum natrium, serum phosphorus, serum calcium, serum parathyroid hormone, serum creatinine, and fasting plasma glucose

**Fig. 2:**
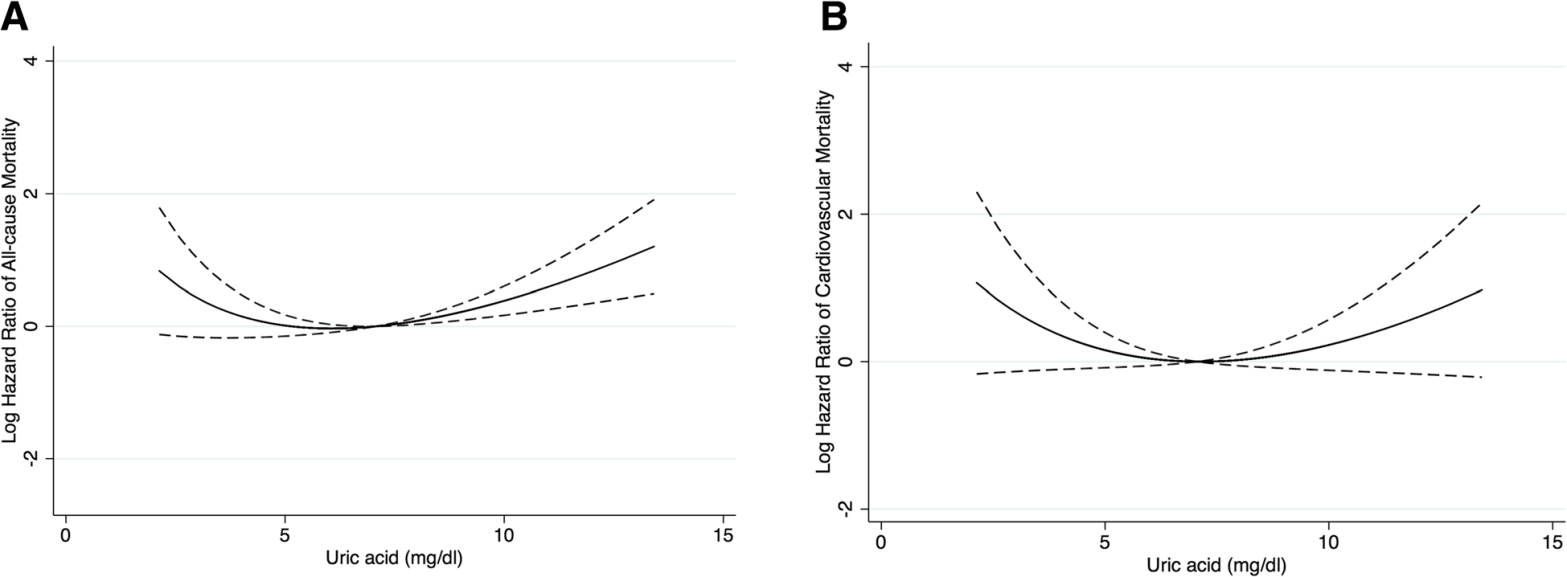
Mortality risk according to uric acid level with the fully adjusted fractional polynomial regression models: (**a**) All-cause mortality and (**b**) cardiovascular mortality. Adjusted for age, sex, body mass index, diabetes mellitus, cardiovascular disease, residual renal function, hemoglobin, serum albumin, serum potassium, serum natrium, serum phosphorus, serum calcium, serum parathyroid hormone, serum creatinine, and fasting plasma glucose

Furthermore, these associations were confirmed by using fractional polynomial regression analyses, where UA concentrations were modeled as a continuous variable. Figure 2 summarizes the results. Different from a wide range of CIs of the all-cause mortality risk for lower UA levels without reaching statistical significance, an increase in the all-cause mortality risk was more prominent in the higher UA levels with a narrow CI range. Similar to the results of multivariable Cox model, there was also no significant association between cardiovascular mortality and serum UA level. Therefore, we subsequently performed survival analyses by stratifying the UA into 2 groups: UA < 7.28 mg/dL and UA ≥7.28 mg/dL.

### Subgroup analyses

**Fig. 3:**
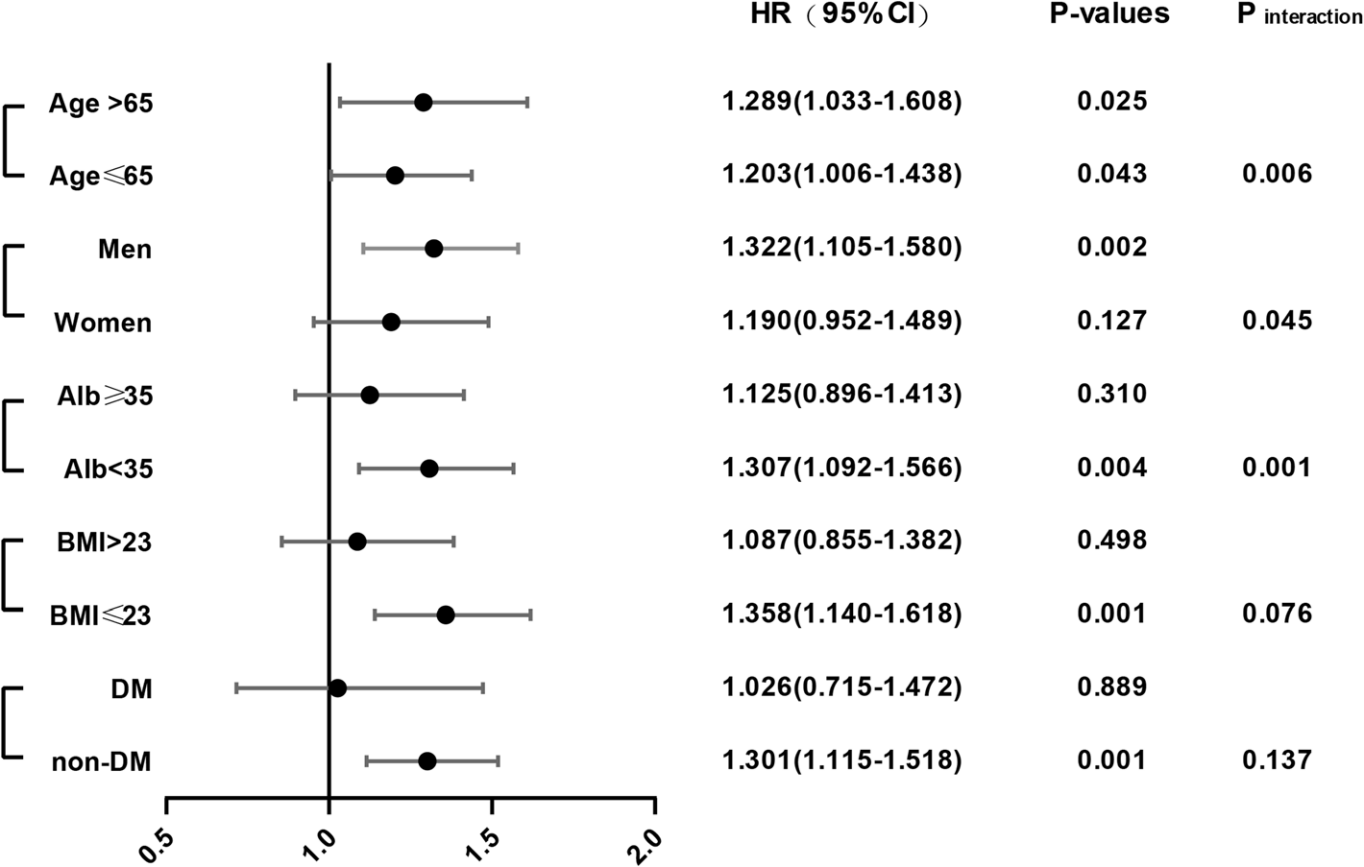
Stratification analyses. A comparison of the adjusted hazard ratios of all-cause mortality for the subgroups is presented by forest plot. Adjusted for age, sex, body mass index, diabetes mellitus, cardiovascular disease, residual renal function, hemoglobin, serum albumin, serum potassium, serum natrium, serum phosphorus, serum calcium, serum parathyroid hormone, serum creatinine, and fasting plasma glucose for each subgroup (excluding its own group)

Based on the results of *P* value of interaction, we further divided patients into subgroups, according to traditional risk factors, including age, sex, BMI, albumin, and diabetes. The adverse effect of higher UA level (≥7.28 mg/dL) on all-cause mortality was more prominent in men group (adjusted HR, 1.322; 95% CI, 1.105–1.580; *P* = 0.002), hypoalbuminemia group (Albumin < 35) (adjusted HR 1.307, 95% CI, 1.092–1.566; *P* = 0.001), normal weight group (BMI ≤23 kg/m2) (adjusted HR 1.358, 95% CI, 1.140–1.618; P = 0.001), and patients without DM (adjusted HR 1.301; 95% CI, 1.115–1.518; P = 0.001), while the effect was similar between patients with age > 65 years (adjusted HR, 1.289; 95% CI, 1.033–1.608; *P* = 0.025) and age ≤ 65 years (adjusted HR, 1.203; 95% CI, 1.006–1.438; *P* = 0.043) (Fig. 3). For cardiovascular mortality, there were no significant differences between all the subgroups (Additional file 3: Figure S3).

## Discussion

In this large multicenter observational study, we explored the associations between serum UA and all-cause and cardiovascular mortality in PD patients. Using data from the ZJRDS of ESRD, we found that, among Chinese PD population, serum UA level was affected by sex, nutritional status indicators, such as BMI, albumin and phosphorus, as well as other laboratory values of potassium, calcium and fasting plasma glucose. The major finding of this study is that a higher UA level (≥7.28 mg/dL) was significantly associated with higher risk of all-cause mortality, independent of several confounding factors. This observation was further confirmed by similar results noted when UA was analyzed with fractional polynomial regression models as a continuous variable. Though a lower serum UA level (≤6.67 mg/dL) was associated with increased all-cause mortality risk in a Kaplan-Meier survival analysis, this effect disappeared after adjustment for potential confounders (eg, demographic characteristics, laboratory values, and comorbidity).

Only a handful of studies have described the relationship between serum UA and mortality among patients on peritoneal dialysis. To date, whether elevated serum UA level is an independent risk factor for mortality has been a matter of some debate. A prospective study in Sweden found that serum UA showed a J-shaped relationship with all-cause mortality [12]. Another 2 previous studies from China demonstrate that the a higher UA level leaded a worse effect and was associated with increased mortality in PD patients [17, 18]. Furthermore, another large investigation of 1278 PD patients found that elevated serum UA was an independent predictor of cardiovascular mortality in diabetic male PD patients, not in the female [22]. In addition, an U-shape UA level-mortality relationship was reported in incident PD patients [20]. Similar to the results of these previous studies, our results showed that a higher UA level (≥7.28 mg/dL) resulted in a poorer outcome and was an independent risk factor for all-cause mortality in PD patients.

The underlying mechanisms connecting hyperuricemia with higher HR of mortality in patients treated with PD are still far from being well understood. Several possible explanations were proposed to elucidate the detrimental effects of UA. First, experimental and clinical studies have provided increasing evidence that UA is an endothelial toxin and plays a role in endothelial dysfunction. Experimentally induced hyperuricemia was shown to be related to the inhibition of nitric oxide production [27], which activated the renin-angiotensin system and subsequently resulted in endothelial impairment [28, 29]. Kang DH et al. demonstrated that UA altered cell proliferation and migration, as well as nitric oxide release in vascular endothelial and smooth muscle cells via activation of an inflammatory pathway involving p38 [30]. Another inflammatory mediator, monocyte chemoattractant protein-1(MCP-1), was found to be involved in the UA-induced macrophage infiltration in atherosclerotic vessels [31]. Interventional trials showed that allopurinol, a widely used UA-lowering drug, normalized endothelial dysfunction in non-CKD [32, 33] and CKD patients [34]. Second, serum UA levels were associated with CRP levels, which was reported in CKD patients before dialysis treatment [35]. As shown above, uric acid also was a proinflammatory factor that stimulated MCP-1 and p-38, and subsequently induced CRP. Higher CRP level was highly predictive of vascular disease and mortality in dialysis patients [36]. This mechanism may also account for the association between hyperuricemia and increased mortality in PD patients. In addition, elevated UA levels have been shown to be related with rheological disturbances, such as impaired platelet aggregation and adhesiveness [4, 37]. Finally, the impact of hyperuricemia on the RRF may be another explanation. For example, A Korean study including 134 incident PD patients with residual urine volumes>200 mL/ d during a follow-up period of 24 months showed that hyperuricemia significantly caused an accelerated decline in residual kidney function [38], which is a strong predictor of patient survival in PD patients [39]. Another study showed an U-shaped relationship between UA levels and the rate of RRF decline in CAPD patients, with a faster decline rate in those of higher and lower UA groups [40].

It is interesting that there was no significant association between UA and RRF after adjusted with various covariates, though in unadjusted model, RRF significantly affected the serum UA level, as shown in Additional file 4: Table S1. In CKD patients, since uric acid is excreted primarily by the kidney, and hence a decrease in the RRF along with the CKD progression is inevitably accompanied by a rise in the serum UA level, with approximately half of the subjects becoming hyperuricemic by the time dialysis is initiated [12]. Conversely, an elevated UA has almost uniformly been found to be strongly associated with the development of CKD [41], but not always with the progression of CKD [10, 42]. These studies showed that UA was not an independent predictor for CKD progression [10, 42], which was similar with our data in Additional file 4: Table S1. The possible explanation for the findings is that UA may not be such an important contributor to progression in established kidney disease especially in the presence of powerful risk factors for progression of CKD such as proteinuria and high blood pressure [10].

Our present study showed that a lower serum UA (≤6.67 mg/dL) had no significant influence on increasing all-cause mortality in multivariable Cox analysis after adjusting for various covariates, though a lower one was associated with increased all-cause mortality risk in a Kaplan-Meier survival analysis, with the lowest risk in the 3 middle quintile. The result was a little different from Suliman’s [12] or Chang’s [20] study which found a J-shape or U-shape relationship respectively, and opposite of Lai’s [19] study which showed an inverse relationship. There are a few potential explanations for contradictory results. First, although all these studies and our study were designed retrospectively, the included population of our study was much larger than the others, which may cause statistical discrepancy. Second, the patients analyzed in Suliman’s study were undergoing not only PD but also HD [12]. And higher BMI and proportion of DM were found in Suliman’s and Chang’s studies [12, 20]. Third, the patients enrolled in Lai’s study were older, with a lower RRF and with a lower hemoglobin level [19]. All these factors were associated with increased risk of mortality, which may result in different result from our study. Additionally, the adjusted parameters added into the analysis model were inconsistent between these studies and our study. In our study, lower serum UA level is independently associated with lower serum albumin, lower BMI, lower phosphorus concentrations, which may be evidence of worse nutritional status. However, after considering the influence of mixed factors, such as age, sex, prevalence of diabetes, serum creatinine level, calcium level, the detrimental effect of lower UA level may be overwhelmed by the beneficial effects. The mechanism underlying the paradoxical association between UA and all-cause mortality among PD patients has not been well clarified and is multifactorial in nature.

In our present study, the PD patient group with lower and higher UA level increased cardiovascular mortality, but after adjusting for uremia-related and traditional cardiovascular risk factors, this relationship was no longer significant. Similarly, two other studies on HD patients reported that serum UA level was correlated with all-cause mortality but not cardiovascular mortality [15, 43]. Another multicenter study of 2264 patients on PD patients also showed that the prognostic value of UA for cardiovascular mortality weakened or disappeared after adjustment of the models [21]. The inconsistent of study results relating UA to cardiovascular mortality may due to its dual effects on cardiovascular outcomes. Excess UA turned out to be closely related to endothelial dysfunction, oxidative stress, inflammation and activation of the renin-angiotensin system, which may lead to cardiovascular diseases. On the other hand, both in vitro and in vivo experiments have shown UA to be a powerful free radical scavenger which could benefit the cardiovascular system [44, 45]. Therefore, the final trend for the UA level-cardiovascular mortality relationship for a specific population might depend on the balance between the protective and toxic effects of UA.

Another interesting finding from our data was that the adverse effect of higher UA level on all-cause mortality was more prominent in male patients. Although in the general population, men have higher serum UA levels than women, whether serum UA level has a sex-related difference on mortality has been uncertain. Such a sex-related difference had been indicated in recent studies of PD population [18, 19, 22]. There were two studies showed a similar result with our study that higher serum UA concentration was an independent risk factor for mortality in male population [18, 22]; while another observed this relationship only in women [19]. The inconsistent results may be explained by the differences in the populations studied, follow-up periods, adjusted confounders and serum UA levels between men and women. Serum UA level has been reported to be related to menopausal status in women [46], which may also influence the impact of UA on clinical outcomes. In our study, the mean serum UA levels in women younger than 45 years (*n* = 1270, 6.93 ± 1.15 mg/dL) were significantly higher than those 45 years or older (*n* = 2969, 6.82 ± 1.20 mg/dL) (*P* = 0.007). Then we separately examined the relationship between UA level and all-cause mortality in the two different age groups and found no significant association (data not shown). The potential mechanisms of the sex-related differences in association of UA level-mortality are still poorly understood.

The strengths of our study are as follows: (1) the major one was that our study included a large number of patients treated with PD in multiple centers with a long-term follow-up about 10 years, representing nearly 10% of the total PD patient population in China; (2) the data were collected using carefully standardized methods and multiple baseline laboratory measurements were averaged, leading to more reliable data; (3) the association between serum UA level and mortality was examined for both overall patients and by several important confounders of serum UA including age, sex, albumin level, BMI, and diabetes; (4) we completed a detailed assessment using both traditional Cox regression model and fractional polynomial regression model, and our results remained robust after several sensitive analysis and adjustments for multiple potential confounders.

However, several limitations should be noted. First of all, the treatment of PD can lower and clear serum UA through peritoneal diffusion and convective, thus serum UA level varies by the PD treatment in the follow-up period [47]. In our study, only baseline data were used in analysis, which did not cover the detail longitudinal changes in serum UA levels during the observation period. Second, due to the retrospective nature of the study, despite our best efforts to adjust for significant confounding factors, residual confounding factors cannot be completely excluded. For example, some potentially important characteristics which could affect the serum UA level and patient survival, such as CRP, SGA (subjective global assessment) scores, ferritin, UA-lowering agents, dietary intakes such as seafood and red meat, lifestyles, hypertension, PD adequacy-related parameters such as D/P creatinine and total weekly Kt/V were not examined. Moreover, the mortality rate of our study may have been underestimated due to the voluntary nature of registration in the Renal Data system, with submission of death reports and causes of death being easily missed. Last, all of our PD patients included in this study were mainly from China, which means results may not apply to patients from other geographic areas or ethnic populations. Despite these limitations, we believe this study contributes to the rather limited knowledge of the predictive value of UA in PD patients.

## Conclusion

In summary, our study demonstrated that higher serum UA level was strongly associated with a higher risk of all-cause mortality, except for cardiovascular mortality, in a large cohort of Chinese PD patients. This association was more prominent in male, relatively lower serum albumin and BMI, non-DM patients. Our results provide evidence regarding the treatment of hyperuricemia in the PD population. Further studies are needed not only to determine whether UA-lowering treatment improve survival in PD patients, but also to elucidate the underlying mechanisms.

## Additional files

Additional file 1: **Figure S1**. Flow chart of study enrollment. (PDF 11 kb)

Additional file 2: **Figure S2**. Distribution of serum uric acid concentrations (*n* = 9,405). (PDF 58 kb)

Additional file 3: **Figure S3**. Stratification analyses. (PDF 182 kb)

Additional file 4: **Table S1**. Baseline parameters affected serum uric acid levels. (PDF 61 kb)

Additional file 5: **Table S2**. Hazard ratios of baseline parameters for all-cause mortality risk factors. (PDF 55 kb)

Additional file 6: **Table S3**. Hazard ratios of baseline parameters for cardiovascular mortality risk factors. (PDF 55 kb)

## Data Availability

The datasets used and/or analysed during the current study are available from the corresponding author on reasonable request.

## Abbreviations

AKP: alkaline phosphatase
BMI: body mass index
CKD: chronic kidney disease
CVD: cardiovascular disease
DM: diabetes mellitus
ESRD: end-stage renal disease
FPG: fasting plasma glucose
HD: hemodialysis
HR: hazard ratios
MCP-1: monocyte chemoattractant protein-1
PD: peritoneal dialysis
PTH: parathyroid hormone
RRF: residual renal function
UA: Uric acid
ZJRDS: Zhejiang Renal Data system
