# Supplementary material for: High serum uric acid level is a mortality risk factor in peritoneal dialysis patients: a retrospective cohort study
Source: Nutr Metab (Lond). 2019 Aug 1;16:52. doi: 10.1186/s12986-019-0379-y (PMC6670192; doi:10.1186/s12986-019-0379-y)
Supplement: Supplementary file 1 — Figure S1. Flow chart of study enrollment. (PDF 11 kb) [file 12986_2019_379_MOESM1_ESM.pdf]

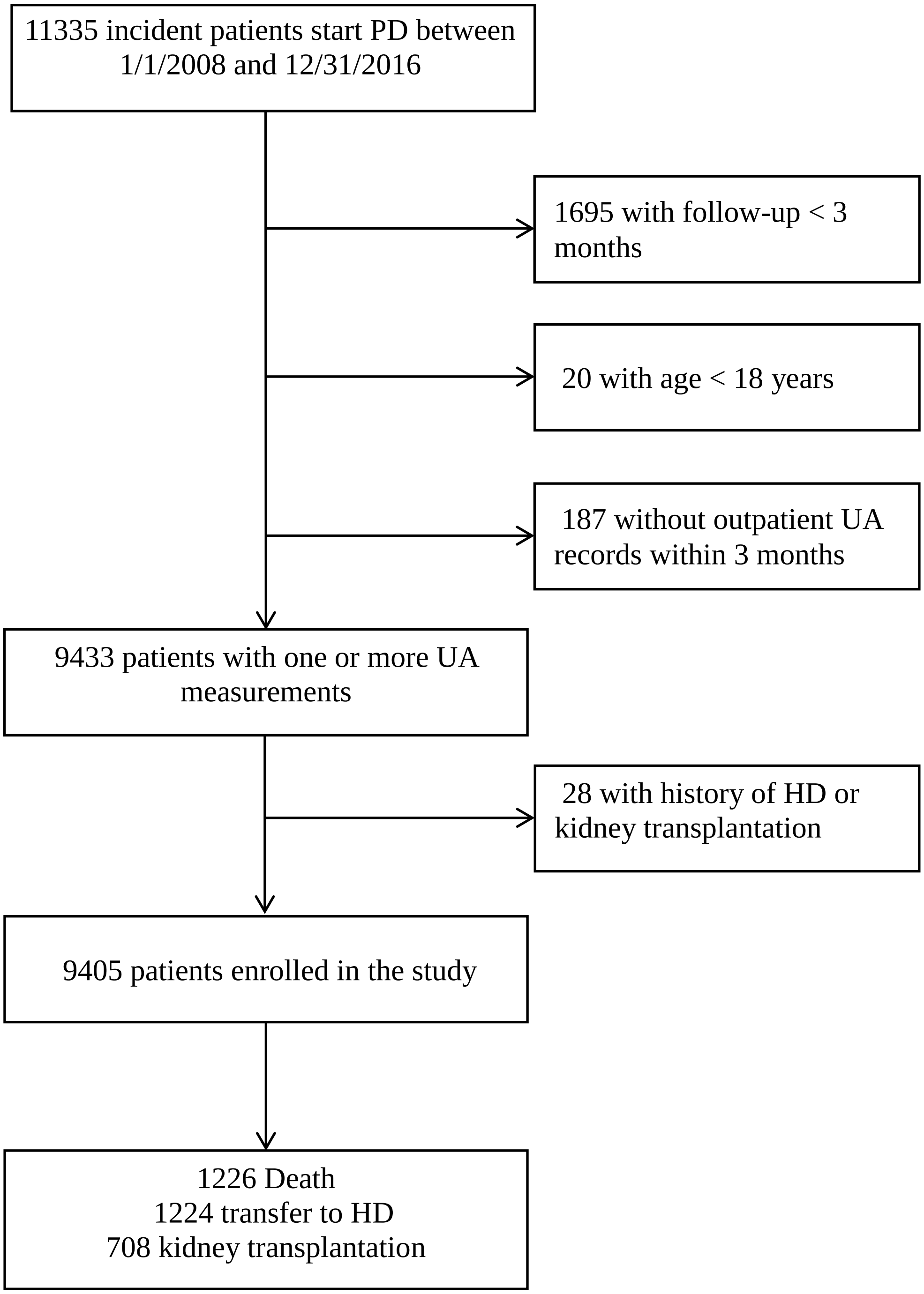

Supplementary Figure S1. Flow chart of study enrollment. Between January 2008 and December 2016, a total of 9405 peritoneal dialysis patients were included.
