# Supplementary material for: High serum uric acid level is a mortality risk factor in peritoneal dialysis patients: a retrospective cohort study
Source: Nutr Metab (Lond). 2019 Aug 1;16:52. doi: 10.1186/s12986-019-0379-y (PMC6670192; doi:10.1186/s12986-019-0379-y)
Supplement: Supplementary file 2 — Figure S2. Distribution of serum uric acid concentrations (n = 9,405). (PDF 58 kb) [file 12986_2019_379_MOESM2_ESM.pdf]

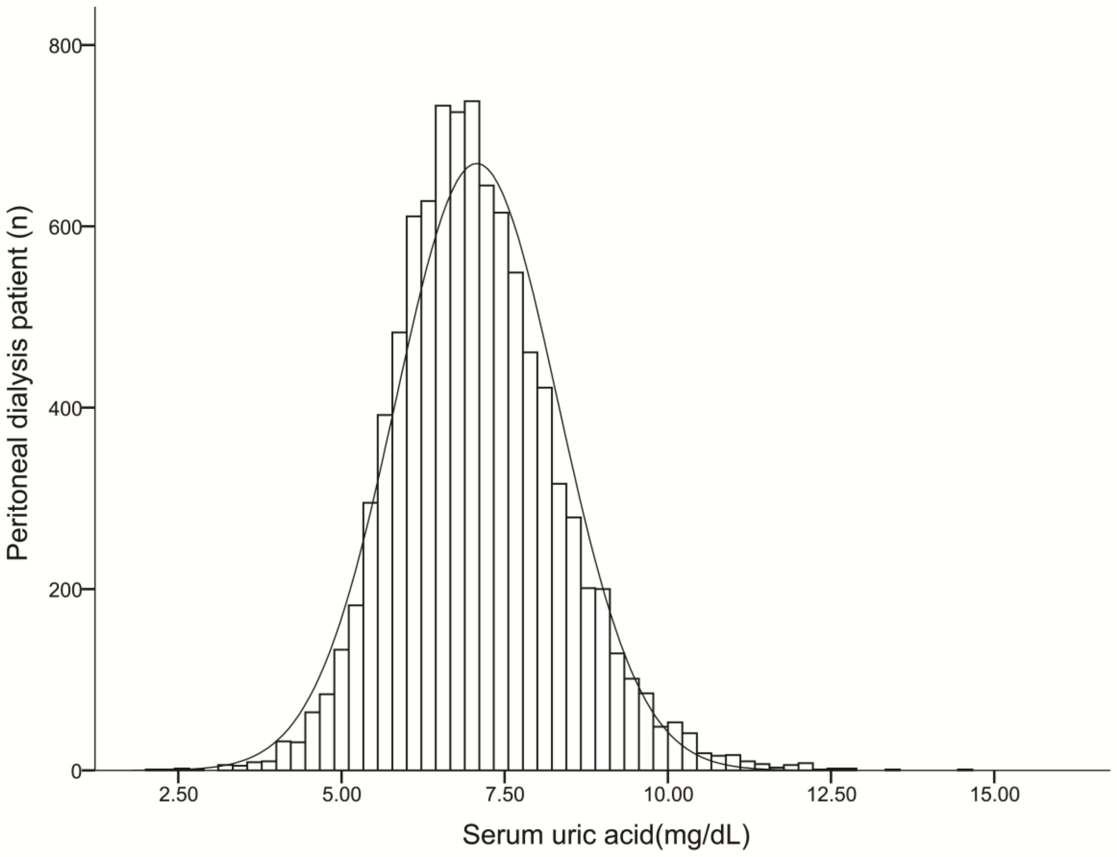

Supplementary Figure S2. Distribution of serum uric acid concentrations (n = 9,405). Mean, 7.07 mg/dL; standard deviation, 1.246 mg/dL.
