# Supplementary material for: High serum uric acid level is a mortality risk factor in peritoneal dialysis patients: a retrospective cohort study
Source: Nutr Metab (Lond). 2019 Aug 1;16:52. doi: 10.1186/s12986-019-0379-y (PMC6670192; doi:10.1186/s12986-019-0379-y)
Supplement: Supplementary file 3 — Figure S3. Stratification analyses. (PDF 182 kb) [file 12986_2019_379_MOESM3_ESM.pdf]

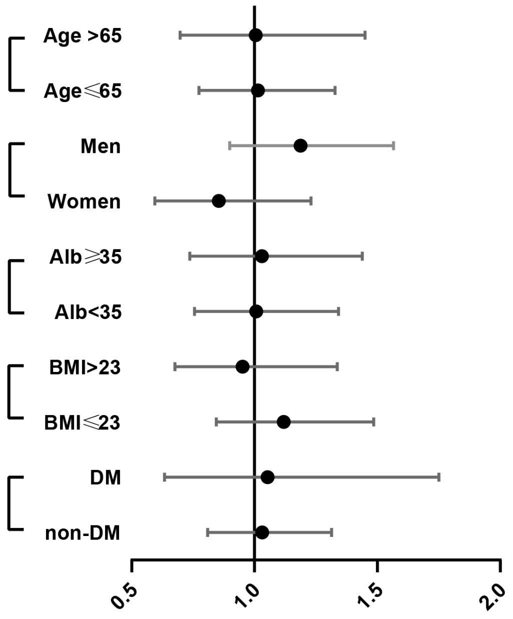


| | HR（95%CI） | P-values | P interaction |
|---|---|---|---|
| Age >65 | 1.005(0.697-1.450) | 0.978 | |
| Age≤65 | 1.014(0.774-1.328) | 0.922 | 0.006 |
| Men | 1.187(0.899-1.566) | 0.226 | |
| Women | 0.855(0.594-1.230) | 0.398 | 0.045 |
| Alb≥35 | 1.030(0.737-1.439) | 0.862 | |
| Alb<35 | 1.007(0.756-1.342) | 0.961 | 0.001 |
| BMI>23 | 0.951(0.676-1.337) | 0.771 | |
| BMI≤23 | 1.119(0.844-1.485) | 0.434 | 0.076 |
| DM | 1.053(0.634-1.750) | 0.841 | |
| non-DM | 1.031(0.809-1.314) | 0.806 | 0.137 |

**Supplementary Figure S3.** Stratification analyses. A comparison of the adjusted hazard ratios of cardiovascular mortality for the subgroups is presented by forest plot. Adjusted for age, sex, body mass index, diabetes mellitus, cardiovascular disease, residual renal function, hemoglobin, serum albumin, serum potassium, serum natrium, serum phosphorus, serum calcium, serum parathyroid hormone, serum creatinine, and fasting plasma glucose for each subgroup (excluding its own group).
