# Supplementary material for: High serum uric acid level is a mortality risk factor in peritoneal dialysis patients: a retrospective cohort study
Source: Nutr Metab (Lond). 2019 Aug 1;16:52. doi: 10.1186/s12986-019-0379-y (PMC6670192; doi:10.1186/s12986-019-0379-y)
Supplement: Supplementary file 4 — Table S1. Baseline parameters affected serum uric acid levels. (PDF 61 kb) [file 12986_2019_379_MOESM4_ESM.pdf]

Supplementary Table S1. Baseline parameters affected serum uric acid levels.

Variants	Serum uric acid level			
	Unadjusted		Adjusted	
	B	P value	B	P value
Age (yr)	-0.009	<0.001	-0.004	0.001
Sex (ref. men)	-0.399	<0.001	-0.341	<0.001
BMI (kg/m^2)	0.053	<0.001	0.034	<0.001
Hemoglobin (g/dL)	-0.004	<0.001	0.001	0.435
Albumin (g/dL)	0.028	<0.001	0.024	<0.001
Baseline creatinine (mg/dL)	0.051	<0.001	-0.013	0.021
Calcium (mmol/L)	-0.439	<0.001	-0.296	0.001
Phosphorus (mmol/L)	0.869	<0.001	0.852	<0.001
Potassium (mmol/L)	0.314	<0.001	0.033	0.301
Natrium (mmol/L)	0.045	<0.001	0.023	0.001
PTH (pg/mL)	0.000	<0.001	0.000	0.551
RRF (mL/min/1.73m^2)	0.000	<0.001	0.000	0.496
FPG (mmol/L)	-0.064	<0.001	-0.035	<0.001
DM(yes)	-0.169	<0.001	-0.077	0.094
CVD (yes)	-0.077	0.189	-0.042	0.481
Follow-up duration(mo)	-0.005	<0.001	-0.005	<0.001

Note: B, regression coefficient with serum uric acid levels. Adjusted for age, sex, BMI, hemoglobin, albumin, Baseline creatinine, calcium, phosphorus, Potassium, Natrium, PTH, RRF, FPG, DM, CVD, Follow-up duration.

Abbreviations: BMI, body mass index; PTH, Parathyroid hormone; RRF, Residual renal function; FPG, fasting plasma glucose; DM, diabetes mellitus; CVD, cardiovascular disease.
