# Supplementary material for: High serum uric acid level is a mortality risk factor in peritoneal dialysis patients: a retrospective cohort study
Source: Nutr Metab (Lond). 2019 Aug 1;16:52. doi: 10.1186/s12986-019-0379-y (PMC6670192; doi:10.1186/s12986-019-0379-y)
Supplement: Supplementary file 5 — Table S2. Hazard ratios of baseline parameters for all-cause mortality risk factors. (PDF 55 kb) [file 12986_2019_379_MOESM5_ESM.pdf]

Supplementary Table S2. Hazard ratios of baseline parameters for all-cause mortality risk factors.

Variants	Univariate		
	B	Hazard ratio (95% CI)	P value
Age (yr)	0.062	1.064(1.059-1.069)	<0.001
Sex (ref. men)	0.081	1.085(0.969-1.214)	0.156
BMI (kg/m^2)	0.014	1.015(0.995-1.034)	0.147
Hemoglobin (g/dL)	-0.032	0.969(0.964-0.973)	<0.001
Albumin (g/dL)	-0.108	0.897(0.888-0.906)	<0.001
Baseline creatinine (mg/dL)	-0.138	0.871(0.856-0.887)	<0.001
Calcium (mmol/L)	-2.514	0.081(0.061-0.107)	<0.001
Phosphorus (mmol/L)	-0.823	0.439(0.368-0.523)	<0.001
Potassium (mmol/L)	-0.602	0.548(0.480-0.624)	<0.001
Natrium (mmol/L)	-0.127	0.880(0.860-0.901)	<0.001
PTH (pg/mL)	-0.002	0.998(0.998-0.998)	<0.001
RRF (mL/min/1.73m^2)	0.070	1.073(1.054-1.092)	<0.001
FPG (mmol/L)	0.151	1.163(1.136-1.189)	<0.001
DM(yes)	0.578	1.783(1.530-2.078)	<0.001
CVD (yes)	0.625	1.869(1.539-2.269)	<0.001

Note: B, regression coefficient.

Abbreviations: BMI, body mass index; PTH, Parathyroid hormone; RRF, Residual renal function; FPG, fasting plasma glucose; DM, diabetes mellitus; CVD, cardiovascular disease.
