# Supplementary material for: High serum uric acid level is a mortality risk factor in peritoneal dialysis patients: a retrospective cohort study
Source: Nutr Metab (Lond). 2019 Aug 1;16:52. doi: 10.1186/s12986-019-0379-y (PMC6670192; doi:10.1186/s12986-019-0379-y)
Supplement: Supplementary file 6 — Table S3. Hazard ratios of baseline parameters for cardiovascular mortality risk factors. (PDF 55 kb) [file 12986_2019_379_MOESM6_ESM.pdf]

Supplementary Table S3. Hazard ratios of baseline parameters for cardiovascular mortality risk factors.

Variants	Univariate		
	B	Hazard ratio (95% CI)	P value
Age (yr)	0.054	1.055(1.048-1.063)	<0.001
Sex (ref. men)	0.088	1.092(0.918-1.300)	0.318
BMI (kg/m^2)	0.056	1.057(1.028-1.087)	<0.001
Hemoglobin (g/dL)	-0.030	0.970(0.963-0.977)	<0.001
Albumin (g/dL)	-0.106	0.900(0.886-0.914)	<0.001
Baseline creatinine (mg/dL)	-0.122	0.885(0.861-0.910)	<0.001
Calcium (mmol/L)	-2.508	0.081(0.053-0.126)	<0.001
Phosphorus (mmol/L)	-0.475	0.622(0.478-0.808)	<0.001
Potassium (mmol/L)	-0.337	0.714(0.584-0.872)	0.001
Natrium (mmol/L)	-0.114	0.893(0.860-0.927)	<0.001
PTH (pg/mL)	-0.002	0.998(0.998-0.999)	<0.001
RRF (mL/min/1.73m^2)	0.082	1.085(1.057-1.114)	<0.001
FPG (mmol/L)	0.172	1.188(1.151-1.226)	<0.001
DM(yes)	0.777	2.175(1.743-2.715)	<0.001
CVD (yes)	1.004	2.730(2.108-3.535)	<0.001

Note: B, regression coefficient.

Abbreviations: BMI, body mass index; PTH, Parathyroid hormone; RRF, Residual renal function; FPG, fasting plasma glucose; DM, diabetes mellitus; CVD, cardiovascular disease.
